# Geriatric urolithiasis in the emergency department: risk factors for hospitalisation and emergency management patterns of acute urolithiasis

**DOI:** 10.1186/1471-2369-13-117

**Published:** 2012-09-24

**Authors:** Spyridon Arampatzis, Gregor Lindner, Filiz Irmak, Georg-Christian Funk, Heinz Zimmermann, Aristomenis K Exadaktylos

**Affiliations:** 1 Department of Nephrology and Hypertension, Inselspital, University of Bern, Bern, Switzerland; 2 Department of Emergency Medicine, Inselspital, University of Bern, Bern, Switzerland; 3 Department of Respiratory and Critical Care Medicine and Ludwig Boltzmann Institute for Chronic Obstructive Pulmonary Disease, Otto Wagner Hospital, Vienna, Austria

**Keywords:** Urolithiasis, Geriatric patients, Emergency department, Hospitalisation

## Abstract

**Background:**

Urolithiasis is one of the most common conditions seen in emergency departments (ED) worldwide, with an increasing frequency in geriatric patients (>65 years). Given the high costs of emergency medical urolithiasis treatment, the need to optimise management is obvious. We aimed to determine risk factors for hospitalisation and evaluate diagnostic and emergency treatment patterns by ED physicians in geriatric urolithiasis patients to assist in optimising treatment.

**Methods:**

After receiving ethics committee approval, we examined the records of emergency urolithiasis admissions to our ED between January 2000 and December 2010 to determine risk factors for hospitalisation and to evaluate current diagnostic and emergency treatment patterns in geriatric urolithiasis patients.

**Results:**

1,267 consecutive patients at least 20 years of age with confirmed urolithiasis (1,361 ED visits) and complete follow-up data were analyzed. Geriatric patients comprised 10% of urolithiasis patients with more than half of them experiencing their first urolithiasis episode at ED admission. Although stone site, side and size did not significantly differ between groups, urinary stone disease was more severe in the elderly. The risk of severe complications correlated with increasing age, female sex and diabetes mellitus. Geriatric patients had a two-fold greater likelihood of being hospitalised. A significantly lower percentage of geriatric patients received combined analgesic therapy for pain management (37% vs. 64%, p = <0.001) and supportive expulsive treatment (9% vs. 24%, p = <0.001).

**Conclusion:**

Geriatric patients with urolithiasis have a higher morbidity than younger patients and may be undertreated concerning analgetic and expulsive treatment in ED.

## Background

Since the time of Hippocrates, urolithiasis has presented a challenge for clinicians and is still one of the most common conditions seen in emergency departments (ED) worldwide, with an estimated lifetime risk of 15–25% [1].

It is more frequent in adults, with a peak between 40 and 50 years of age, and is increasingly being seen in geriatric patients [2]. Individuals over the age of 65 years are the fastest growing demographic group. According to current demographic statistics from the Swiss Federal Statistical Office, the proportion of the population aged 65 years or older will have risen from 15% in 2000 to 28% in 2050 [3]. Because of this and the increasing prevalence of nephrolithiasis as a result of environmental and metabolic factors, the numbers at risk are expected to rise further [4,5].

Because ED physicians are often the first to treat urolithiasis, it is important to understand the way they diagnose and evaluate acute urolithiasis treatment patterns in such patients.

Given the high cost of urgent medical urolithiasis treatment in geriatric patients with pre-existing comorbidities, the need to focus on optimal management of an acute stone event is obvious. We nevertheless felt that it would be of benefit to explore the relationship between urolithiasis, factors influencing treatment and referral, and patient age [6-8], and conducted this single-centre retrospective study based on emergency urolithiasis admissions to our ED over the past 11 years. The aim was to determine risk factors for hospitalisation, and to evaluate current diagnostic and treatment patterns of geriatric urolithiasis patients in the ED setting to enable us to optimise patient care.

## Methods

### Setting

Our ED is the only Level I centre in a catchment area serving about 1.8 million people and treats more than 30,000 cases per year. Despite slight variations in clinical practice between the physicians in our ED, the practical evaluation of patients with suspected urolithiasis generally follows the same pattern. Based on actual recommendations, the diagnostic and therapeutic management is at the discretion of the attending emergency physician. Emergency urologist consultations in ED are considered mostly for patients presenting with severe complications.

### Ethical considerations

The Cantonal Ethics Committee (KEK) of Bern approved this study. Data were collected, anonymised, stored, analysed and shared according to the ethics committee standards.

### Data collection and retrospective survey

Consecutive patients with urolithiasis, at least 20 years of age, admitted to our ED between 1 January 2000 and 31 December 2010 were identified using the appropriate search string in the diagnosis or imaging field of our computerised patient database (Qualicare Office, Medical Database Software, Qualidoc AG, Bern, Switzerland).

All patients presenting to the ED with symptoms of renal colic during the study period were initially eligible for study inclusion. Since this medical database allows instantaneous retrieval of past diagnostic reports, discharge summaries, consultations and other relevant medical documents or radiographs, the authors were able to retrospectively analyze the diagnostic results, and therapeutic procedures initiated in the ED. Entry criteria included confirmed urolithiasis by the passage of a stone during admission or the finding of a stone on imaging (using non-contrast-enhanced spiral abdominal computed tomography, standard abdominal computed tomography or abdominal sonography, i.v. urography, or plain abdominal X-ray) performed by attending radiologists in the ED. For patients with multiple stones identified on imaging, the location of the lowest obstructing stone was recorded. Exclusion criteria were the absence of documented imaging techniques in our institution, abdominal sonography as a single diagnostic procedure for stone confirmation performed by a non-certified emergency physician, and age <20 years, since these patients are occasionally admitted and evaluated in the paediatric emergency department.

Following this approach, out of an initial >4,500, 1,500 patients with confirmed urolithiasis were primarily included and classified by age, gender and date of emergency admission. Out of these, 1,361 patients had complete data with relevant medical and urolithiasis history, complete follow-up information from successive ED visits, current medication, discharge and admission status, size, number, side and stone location, and radiological findings, and were included in the analysis.

### Assessment of acute urolithiasis complications based on diagnostic reports, discharge summaries and radiological findings

All acute urolithiasis complications were for the purposed of this investigation graded as: Grade 0 (no severe complications such as asymptomatic presentation); Grade 1 (mild to moderate dilatation of the ureter); Grade 2 (severe dilatation of the ureter); Grade 3 (urinary tract infection); and Grade 4 (fornix rupture, pyelonephritis, urosepsis).

### Statistics

Between-group comparisons of continuous variables were performed using one-way ANOVA. Categorical variables were compared using the χ^2^ test or Fisher’s exact test.

Multivariable logistic regression analysis was used to explore the association of the various predictors with hospitalisation and complications. ‘No hospitalisation’ and ‘No or non-severe complications’ were defined as reference categories. Covariates were sequentially added to the logistic regression model if they were significant in the univariate analyses (p < 0.2) and if they substantially altered the coefficient for either weaning category at an a priori defined level of 10% [9]. The sequences of covariates considered were based on the strength of the univariate association [9]. Robust standard errors were used to account for data clustering.

Data are presented as means ± SD, medians or proportions, as appropriate. A two-sided p-value < 0.05 was considered statistically significant. Statistical analysis was performed using SPSS (SPSS for Windows release 15.0, Chicago, IL) and STATA (STATA/MP 10.0, College Station, TX).

## Results

**Figure 1:**
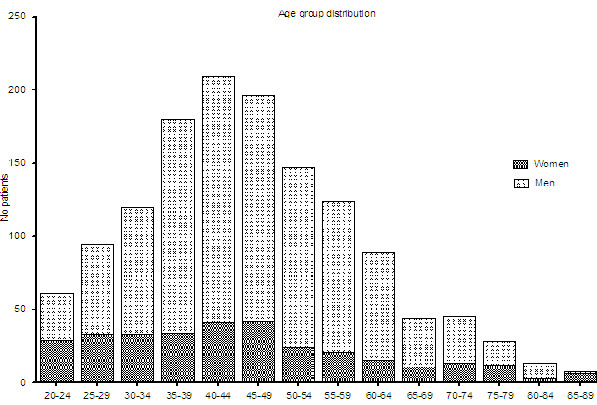
Age distribution of urolithiasis patients admitted to our emergency department.

Out of more than 320,000 ED visits over 11 years, we found 1,361 cases that fulfilled the strict entrance criteria for this study. Table 1 lists patient characteristics. 94/1,267 (7%) patients presenting with urolithiasis were readmitted during the study period; 77% were men. The mean age was 46 ±14 years (median 45, range 20–93), and 90% were younger than 65 years. Age distribution is shown in Figure 1. Based on the evaluation of the first ED admission, 660 (52%) patients had had a previous stone episode. Amongst comorbidities, 167 (13%) were under treatment for hypertension and 33 (3%) had diabetes mellitus. A diagnosis of urolithiasis was confirmed in 1,019 (75%) patients by spiral or abdominal CT, in 721 (53%) by sonography, and in 308 (23%) by intravenous pyelography or plain abdominal radiography. At least two different imaging procedures were used in 572 (42%) cases. Expulsive treatment was administered to 22% of admitted patients. Non-severe complications were present in 1,308 (96%) patients. Severe complications were present in 53 cases (4%). Emergency urological consultations were performed in 529 (39%) cases, and 435 (34%) patients were hospitalised.

**Table 1:** Characteristics of 1,267 patients presenting with urolithiasis to our emergency department between 2000 and 2010

**Variable**	**Number (%) of patients**
ED visits evaluated	1361
Patients with ≥2 ED visits	94 (7)
Men/Women	1042 (77)/318 (23)
Mean age ± SD (median, range)	46 ±14 (45, 20-93)
Patients ≥65 years	141 (10)
Patients <65 years	1220 (90)
Previous history of renal calculi*	660 (52)
Comorbidities	
Hypertension*	167 (13)
Diabetes mellitus*	33 (3)
Imaging	
Stone CT	860 (63)
Abdominal CT	159 (12)
Sonography	721 (53)
KUT radiography/IVP	308 (23)
Patients ≥2 diagnostic procedures	572 (42)
Expulsive treatment*	275 (22)
Severe complications	53 (4)
Urological consultations in ED	529 (39)
Hospitalisation	435 (34)

*only the first ED visit of the patient was evaluated.

Table 2 summarises the characteristics of the geriatric and younger patients. The mean age of the 141 patients ≥65 years was 73 ± 7 years (range 65–93). Geriatric stone formers had a significantly higher female predominance of 33% versus 22% for the younger group (p = 0.001). No statistically significant difference was found regarding allocation of patients who were re-admitted due to recurrent stone episodes during the study (8% versus 7%, p = 0.6), although more geriatric patients were treated for their first symptomatic stone episode (56% versus 43%, p = <0.01). Physicians were found to be more likely to order radiological investigations for younger patients (44% versus 23%, p = <0.01). Comorbidities such as diabetes mellitus (13% versus 2%, p = <0.001), hypertension (49% versus 9%, p = <0.001) and diuretics usage (16% versus 1%, p = <0.001) were significantly more common in geriatric patients.

**Table 2:** Stone characteristics, complications and treatment patterns of patients <65 years of age and ≥65 years

**Variable**	**Number (%) of patients**		**p-value**
	**≥65 years**	**<65 years**	
Patients	141 (10)	1220 (90)	
Mean age ± SD (range)	73±7 (65–93)	43±11 (20–64)	<0.01
Men/Women	94 (67)/46 (33)	948 (78)/272 (22)	0.01
Patients with ≥2 ED visits	11 (8)	83 (7)	0.6
First stone episode	79 (56)	528 (43)	<0.01
Patients ≥2 diagnostic procedures	32 (23)	540 (44)	<0.01
Diabetes mellitus	18 (13)	21 (2)	<0.001
Hypertension	69 (49)	114 (9)	<0.001
Diuretics	22 (16)	11(1)	<0.001
Stone site			0.25
*Kidney*	38 (27)	301 (25)	
*Ureteropelvic junction*	7 (5)	49 (4)	
*Proximal ureter*	15 (11)	124 (10)	
*Middle ureter*	24 (17)	116 (10)	
*Distal ureter*	40 (28)	504 (41)	
*Bladder/passed*	17 (12)	126 (10)	
Stone side			
*Right/left kidney*	50 (36)/47 (33)	427 (35)/466 (38)	0.78
*Bilateral*	27 (19)	201 (17)	
*Bladder/passed*	17 (12)	126 (10)	
Stone size >5mm/≤5mm	42 (35)/79 (65)	310 (36)/551 (64)	0.88
Single/multiple stones	79 (56)/62 (44)	913 (75)/307 (25)	<0.001
Severe complications	13 (9)	40 (3)	0.001
Hospitalisation	65 (46)	394 (32)	0.001
Treatment			
*Analgesics administered in ED*			<0.001
*0*	10 (7)	71 (6)	
*1*	79 (56)	366 (30)	
*≥2 Analgesics*	52 (37)	783 (64)	
*Analgesics*			
*Opioid*	45 (32)	723 (59)	<0.001
*NSAID*	11 (8)	231 (19)	<0.01
*Metamizole*	94 (67)	838 (69)	0.62
*Paracetamol*	36 (26)	327 (27)	0.74
*Expulsive agents*	12 (9)	287 (24)	<0.001
*Antibiotics*	26 (18)	124 (10)	<0.01

**Table 3:** Multivariate logistic regression analysis model for risk factor analysis

**Variable**	**Odds ratio (95 CI)**	**p- value**
**Risk factors for severe complications**		
*Age*	1 .0 (1.00–1.04)	<0.01
*Male vs. female sex*	0.4 (0.2–0.8)	<0.01
*Diabetes mellitus*	8.5 (2.6–27.9)	0.08
*Antibiotic therapy*	29 (13 –64)	<0.0001
*Urological consultation*	0.5 (0.3–1.0)	0.06
**Risk factors for hospitalisation**		
Age ≥65 years	2.0 (1.3–3.0)	0.001
Complication grade		
*0*	Reference	
*1*	2.1 (1.6–2.8)	<0.0001
*2*	3.3 (1.8–6.1)	<0.0001
*3*	1.9 (1.1–3.4)	<0.01
*4*	21.6 (9.5–49.4)	<0.0001
Site		
*Kidney*	1.6 (0.9–2.8)	0.08
*Ureteropelvic junction*	4.1 (1.8–6.1)	<0.0001
*Proximal ureter*	5.3 (2.0–8.4)	<0.0001
*Middle ureter*	3.7 (2.0–6.7)	<0.0001
*Distal ureter*	4.19 (2.5–6.9)	<0.0001
*Bladder/passed stone*	Reference	
Analgesic treatment	1.39 (1.0–1.9)	0.06
Urological consultation	1.25 (0.97–1.62)	0.09

Stone site, side and size did not differ significantly between the two groups, but multiple stones were more frequent in older patients (44% versus 25%, p = <0.001). The severity of urinary stone disease (9% versus 3%, p = 0.001) and the number of hospitalisations (46% versus 32%, p = 0.001) were significantly higher in geriatric patients.

A significantly lower percentage of geriatric patients received a combination of two or more analgesic medications (37% versus 64% p = <0.001). Metamizole was used as first-line analgesic treatment in both groups, but significantly more younger patients also received opiates for pain control (59% versus 32%, p = <0.001), NSAIDs (19% versus 8%, p = <0.01) or expulsive agents (24% versus 9%, p = <0.001). Antibiotic treatment was more frequent in patients ≥65 years (18% versus 10%, p = <0.01).

Multivariate logistic regression analysis in the overall population (Table 3) showed that age and being female were associated with an increased risk of severe complications (odds ratio 1.0, 95% CI, 1.00 to 1.04; odds ratio for male sex 0.4, 95% CI 0.2 to 0.8), as was diabetes mellitus (odds ratio 8.50, 95% CI, 2.6 to 27.9). Geriatric patients had a statistically significant two-fold greater likelihood of being hospitalised (odds ratio 2.0, 95% CI, 1.3 to 3.0; p = 0.001). Hospitalisation strongly correlated with the severity of complications and with stones in the ureter.

## Discussion

We aimed to characterise practice patterns and risk factors for hospitalisation of geriatric urolithiasis patients in an academic teaching hospital ED over an 11-year period to enable us to optimise patient care. Our results show that geriatric patients compromise 10% of urolithiasis admissions to our emergency department, with more than half experiencing their first urolithiasis episode. Although stone site, side and size did not significantly differ between groups, multiple stones were less frequent in younger patients, and the disease was more severe in older patients. The risk of severe complications was higher with advanced age, female sex and diabetes mellitus. Geriatric patients had a two-fold greater likelihood of being hospitalised, and the hospitalisation risk was associated with a greater severity of complications and stones located in the ureter.

Ours is one of the few studies that have examined urolithiasis treatment patterns in the ED, and a new – and surprising – finding was the significantly lower percentage of geriatric patients who received multiple radiological investigations and combined analgesic therapy for pain management and supportive expulsive treatment.

Current gender related epidemiologic findings derived from different geographical regions are indicating a demographic shift, with an increased prevalence of stone disease in female subjects [4,10,11]. The high male prevalence in our study can be attributed to the retrospective design and a possible selection bias. Age-related epidemiological features observed in this study are very similar to those reported in the literature since the incidence of renal colic in elderly persons appears to be between 10 and 12% of urolithiasis patients [6,7]. The true incidence still remains unknown since this estimate is based on hospital admissions, and a significant proportion of patients with urolithiasis are managed as outpatients [12].

Nevertheless, ureteral colic is a significant disease according to emergency physicians, accounting for more than 120,000 hospitalisations during the past 10 years in Switzerland. The majority of patients in our study were discharged from the ED after acute treatment. Admission was only required in cases with complications or those where pain relief could not be achieved. Regarding the diagnostic management of renal stones, spiral CT, which is considered the diagnostic gold standard, was used in 63% of patients. Noteworthy is that our ED physicians ordered more multiple radiological investigations for younger patients.

The increased risk of admission with urolithiasis in patients older than 65 years has several possible explanations. While it may be attributed to a greater severity of disease and accompanying comorbidities, it may also reflect that such patients may be receiving inadequate pain control. Indeed, age per se seems to be a ‘risk factor’ for poor pain management, and there is also growing evidence for an age-related decline in pain sensitivity under experimental conditions [13,14]. Since we did not score pain in our study, we can only postulate that a decrease in pain sensitivity (or higher pain threshold) or a bias on the part of ED providers against adding another drug to a population already at risk for polypharmacy may have accounted for the lower analgesic usage in our patients.Various reports suggested that painful conditions in older adults may be treated suboptimally in the ED setting [15,16].

Several randomised controlled trials and a recent meta-analysis have reported that calcium channel-blockers and alpha-blockers increase the passage rate of ureteral stones [17-19]. In the present study, only 22% of patients with stones discharged from the ED were offered such treatment. Elderly patients in our study were less likely than younger patients to be treated with expulsives (9% versus 24%, p = <0.001). In previous studies examining trends in the prescription of expulsives in EDs in the USA, the overall prevalence of use, given a number-needed-to-treat of 4, was exceedingly low, suggesting a missed opportunity to avoid hospitalisation and urological procedures [18]. We believe that currently physicians in EDs probably prescribe expulsive therapy more frequently than suggested by our findings, which were documented from January 2000 onwards. This is because various publications since then have advocated this approach, and expulsive treatment has been included in guidelines for the treatment of ureter stones since 2007 [20].

Prevention of recurrence through metabolic evaluation after a first stone episode is not cost-effective in younger patients [21]. However, against the backdrop of the higher hospitalisation rate of older patients, a recurrence rate of 50% of cases within 5 years, a decreased thirst sensation, and the rapidly increasing prevalence of nephrolithiasis due to metabolic, demographic and global climate changes, future multi-institutional projects are needed to determine more representative ED practice patterns for urolithiasis management [22,23].

There is great potential for emergency physicians to exert a strong influence on the long-term outcome of patients with stones who are discharged from the ED. It is, of course, unrealistic to expect them to offer comprehensive metabolic and preventative counselling, but it is certainly conceivable that they might routinely give patients basic preventative information. Urologists and physicians in EDs should take this opportunity and develop protocols for the management of cases of acute urolithiasis.

Our trial was limited by its retrospective nature, which may have led to selection bias. However, we used rigorous methods for patient selection. The study was conducted at an academic teaching hospital, and the findings in this patient population may not be generalisable to other settings. Lastly, we do not have information whether patients were directly referred and admitted to the urologists or readmitted to a different hospital after being discharged.

## Conclusion

Geriatric urolithiasis patients are not merely an extension of younger urolithiasis patients. Despite similarities in stone site, side and size, elderly patients have a higher risk of complications and a two-fold greater likelihood of being hospitalised. Adequate analgesia must be assured, and the administration of expulsive agents may represent a key factor in optimum urolithiasis therapy.

## Competing interests

None of the authors has a conflict of interest.

## Authors’ contributions

SA, GL, FI and AKE were involved in study design and data collection. SA, GL, FI, GCF and AKE contributed to the manuscript preparation. GCF and SA contributed to the statistical analysis. HZ and AKE provided oversight of the work and finalised the draft. All authors read and approved the final manuscript.

## Pre-publication history

The pre-publication history for this paper can be accessed here:

http://www.biomedcentral.com/1471-2369/13/117/prepub
